# Optimization of a Method To Quantify Soil Bacterial Abundance by Flow Cytometry

**DOI:** 10.1128/mSphere.00435-19

**Published:** 2019-10-09

**Authors:** Banafshe Khalili, Claudia Weihe, Sarah Kimball, Katharina T. Schmidt, Jennifer B. H. Martiny

**Affiliations:** a Department of Ecology and Evolutionary Biology, University of California, Irvine, Irvine, California, USA; b Center for Environmental Biology, University of California, Irvine, Irvine, California, USA; University of Wisconsin-Madison

**Keywords:** bacterial cell count, ecosystem types, extraction procedure, flow cytometry, soil

## Abstract

Bacterial abundance is a fundamental metric for understanding the population dynamics of soil bacteria and their role in biogeochemical cycles. Despite its importance, methodological constraints hamper our ability to assess bacterial abundance in terrestrial environments. Here, we aimed to optimize the use of flow cytometry (FCM) to assay bacterial abundances in soil while providing a rigorous quantification of its limitations. Soil samples were spiked with Escherichia coli to evaluate the levels of recovery efficiency among three extraction approaches. The optimized method added a surfactant (a tetrasodium pyrophosphate [TSP] buffer) to 0.1 g of soil, applied an intermediate degree of agitation through shaking, and used a Nycodenz density gradient to separate the cells from background debris. This procedure resulted in a high (average, 89%) level of cell recovery. Recovery efficiencies did not differ significantly among sites across an elevation gradient but were positively correlated with percent carbon in the soil samples. Estimated abundances were also highly repeatable between technical replicates. The method was applied to samples from two field studies and, in both cases, was sensitive enough to detect treatment and site differences in bacterial abundances. We conclude that FCM offers a fast and sensitive method to assay soil bacterial abundance from relatively small amounts of soil. Further work is needed to assay differential biases of the method across a wider range of soil types.

**IMPORTANCE** The ability to quantify bacterial abundance is important for understanding the contributions of microbial communities in soils, but such assays remain difficult and time-consuming. Flow cytometry offers a fast and direct way to count bacterial cells, but several concerns remain in applying the technique to soils. This study aimed to improve the efficiency of the method for soil while quantifying its limitations. We demonstrated that an optimized procedure was sensitive enough to capture differences in bacterial abundances among treatments and ecosystems in two field studies.

## Abstract (precis)

The ability to quantify bacterial abundance is important for understanding the contributions of microbial communities in soils, but such assays remain difficult and time-consuming. Flow cytometry offers a fast and direct way to count bacterial cells, but several concerns remain in applying the technique to soils. This study aimed to improve the efficiency of the method for soil while quantifying its limitations. We demonstrated that an optimized procedure was sensitive enough to capture differences in bacterial abundances among treatments and ecosystems in two field studies.

## INTRODUCTION

Bacterial abundance is a fundamental metric for understanding the ecology of soil bacteria. Given the importance of bacteria in terrestrial soil biogeochemistry (1–3), there is a great need to develop fast and reliable methods for quantification of bacterial abundance in soil. Currently, many researchers use indirect measures of activity and metabolism, including quantifying ATP, phospholipid fatty acids (PLFA), and microbial biomass carbon (MBC) as a proxy for bacterial abundance in environmental samples such as soils (4, 5). Alternative methods measure abundance more directly, for instance, by epifluorescence microscopy or by molecular methods such as real-time quantitative PCR (qPCR) (5–8), but these methods are resource- and time-intensive.

Flow cytometry (FCM) offers another direct way to assess soil bacterial abundance. Like microscopy, the method first requires separating the cells from soil particulates; however, the counting step itself is faster and more efficient (more cells counted) (4). While FCM is widely used to count bacteria in the medical sciences and aquatic environments (9–11), it has not typically been applied to soils. Yet recent studies comparing various quantitative measurements suggest that FCM analysis may be a viable option for soil (4, 5, 7, 12). For instance, Bressan et al. (7) reported that FCM counts could distinguish between four crop management systems, with higher bacterial abundances in conventional systems than in organic systems.

Despite these promising results, at least three concerns remain in applying FCM methods to soils. The first is the efficiency of cell extraction from soil matrices (4, 13). Bacterial cells adsorb onto the complex soil matrix, which includes clay, organic matter, and humic acids (14). Thus, both traditional microscopy and FCM require maximizing the extraction of cells from the matrix while minimizing cell damage. Various approaches involve the same two steps: (i) detachment of cells from soil particles (by shaking or sonication in a solution) and (ii) separation of the cells from soil debris (by filtration or centrifugation) before quantification (5, 6, 15). One promising method for the latter step is high-speed centrifugation of soil in a density gradient medium (4, 8, 9). A number of reports have indicated that purification of terrestrial soils or marine sediments in a Nycodenz gradient removes most organic and inorganic particles (4, 6, 14), while allowing high rates of recovery of bacterial cells (6, 9, 16).

A second concern is the ability to distinguish between intact cells and the background noise (autofluorescence) of debris particles (the signal-to-noise ratio) during cell quantification. Efficient separation of the cells from soil debris is important here as well. In both microscopy and FCM, bacterial cells are fluorescently stained to allow quantification. This fluorescent signal can be masked by nonspecific dye binding and autofluorescence of soil particles (17). However, an advantage of flow cytometry is that it can be used to distinguish signal from noise by identifying cells on the basis of size, fluorescence intensity, and wavelength (9). A common gating strategy to distinguish background debris from bacterial cells is to use forward scatter (FSC) and side scatter (SSC) to identify events involving viable single cells; cellular debris and dead cells often have lower levels of forward scatter and higher side scatter than live cells (18). In addition, fluorescence intensity can be useful for further distinguishing between intact and damaged cells (18).

Finally, although all cell quantification methods are subject to bias, a particular concern for methods that require cell extraction is that soil type might introduce biases specific to each soil, making comparisons across locations difficult. In particular, precise estimates of bacterial counts remain a challenge for samples high in organic matter content (4, 13, 19).

Here, we aimed to improve the application of flow cytometry for bacterial cell counts in different types of soils by considering these three concerns. Specifically, we sought to (i) confirm that we could distinguish intact bacterial cells from background particles on the flow cytometer; (ii) compare the results of analyses of detachment of intact bacterial cells from the soil matrix among three extraction procedures; and (3) test the efficiency of the optimal extraction procedure across a range of soil types. To do this, we sampled a variety of soils from sites over a range of physical and chemical characteristics and evaluated levels of cell recovery among the procedures by adding known amounts of cultured cells to the environmental samples. Finally, we applied the optimized method to two field studies to test whether the method was sensitive enough to distinguish cell abundances among treatments and ecosystem types.

## RESULTS

### Flow cytometry protocols.

**FIG 1:**
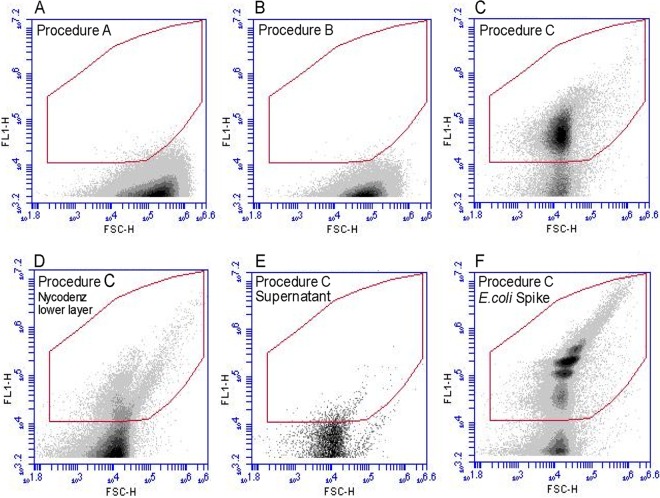
Representative examples of flow cytograms of the different extraction procedures performed on bulk soil samples. Bivariate dot plot diagrams of the intensity of the green fluorescence channel (FL1) versus the forward scatter (FSC-H) are shown. The red polygonal gate defines the expected region of bacterial cells. (A) Procedure A performed using an ultrasonication bath and filtration resulted in few counts within the gate. (B) Procedure B performed using a tissue homogenizer and filtration also resulted in few counts. (C) Procedure C performed using detergent, shaking, and Nycodenz density gradient separation resulted in a distinct population within the gate. (D) The lower layer of the Nycodenz gradient shows few events within the defined gate, demonstrating good separation of the cells. (E) The supernatant of the Nycodenz cell pellet also shows few events. (F) Procedure C spiked with E. coli showed that the cultured cells were localized within the defined gate.

The three cell extraction procedures led to vastly different cell counts in analyses of the same soil samples. Both the ultrasonic and tissue homogenizer treatments led to very low bacterial counts; almost no events were detected within the defined gates (*n* = 29) (Fig. 1A and B). Adjusting the length of centrifugation after sonication did not increase cell counts. This result was confirmed by low recoveries of cells in Escherichia coli*-*spiked samples. We recovered averages of only 21% (*n* = 2) and 5% (*n* = 2) of E. coli cells with procedure A and procedure B, respectively, suggesting that most cells were still bound to the soil matrix and/or were damaged during sonication and homogenizing.

In contrast, procedure C involving detergent (Tween 80), shaking, and density gradient centrifugation resulted in a distinct population of stained intact cells on the cytograms separate from background particles (Fig. 1C). We detected relatively few cells in the lower phase after Nycodenz purification and in the supernatant after the second centrifugation, suggesting good recovery of the detached cells in the purified thin layer on top of the Nycodenz cushion liquid phase (Fig. 1D and E). Indeed, across all soil samples tested (*n *= 15), the spiked E. coli cells showed a large population in the gate (Fig. 1F) and recovery rates were very high (mean = 89% ± 7.7).

**FIG 2:**
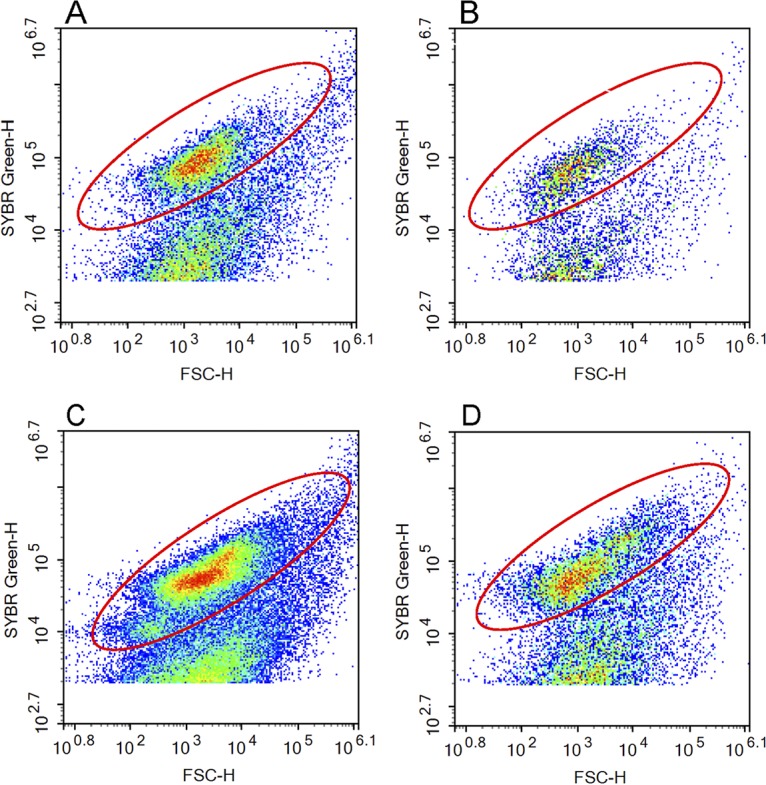
Cytogram of samples from pine-oak litter (A) and bulk soil (B) and scrubland litter (C) and bulk soil (D). The red circle indicates the gate used to count bacterial cells. Warmer colors indicate higher densities of counts.

Although we did not optimize the method on leaf litter samples, we tested whether the bulk soil method could also be used for leaf litter to compare abundances across the sample types. Bacterial cells extracted from leaf litter also formed distinct populations on the cytograms in the same locations as those extracted from bulk soil (Fig. 2). We note that for both leaf litter and soil, using a small amount of initial sample (0.1 g) was sufficient to count a high number of stained cells while reducing the amount of background debris.

**FIG 3:**
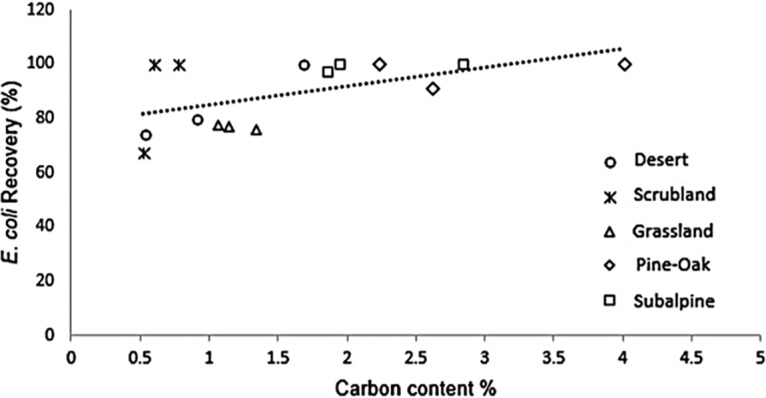
Percent E. coli recovery versus percent carbon content of bulk soil samples from the elevation gradient.

Finally, we compared the efficiency levels of procedure C across a range of bulk soils along an elevation gradient. The physiochemical properties of the soils (pH, percent total carbon and nitrogen, and percent clay content) differed significantly among the five sites (analysis of variance [ANOVA]; for all parameters, *P < *0.05) (Table 1). Despite the differences, the levels of recovery of E. coli cells did not differ across sites (one-way ANOVA; *F *= 2.41, *df *= 4; *P *= 0.118). However, the fraction recovered was positively correlated with percent carbon in the sample (*r *= 0.55; *P < *0.05) (Fig. 3) but not with percent clay content (*r* = −0.21; *P *= 0.44).

**TABLE 1:** Physical and chemical characteristics of soils at five locations along a southern California elevation gradient^*a*^

Ecosystem	pH	% C		% N		% clay content	% E. coli recovery
		Soil	Litter	Soil	Litter		
Desert	6.2 (0.24)	1.1 (0.59)	26 (2.8)	0.08 (0.04)	0.8 (0.2)	9.5 (1.74)	84 (13.6)
Scrubland	8.5 (0.49)	0.6 (0.13)	34 (5.1)	0.05 (0.01)	1.2 (0.2)	5.1 (0.57)	89 (18.7)
Grassland	6.3 (0.10)	1.2 (0.14)	37 (1.7)	0.1 (0.01)	1.7 (0.3)	10.3 (1.98)	77 (0.84)
Pine-Oak forest	6.1 (0.03)	2.9 (0.94)	47 (1.4)	0.12 (0.03)	0.7 (0.1)	9.5 (1.74)	97 (5.2)
Subalpine forest	6.2 (0.02)	2.2 (0.54)	44 (4.0)	0.08 (0.01)	0.7 (0.1)	5.1 (0.57)	99 (1.7)

a Values in parentheses represent 1 standard deviation (*n *= 3).

### Application to two field studies.

The optimized procedure was applied to two field studies, (i) a survey comparing bulk soil and leaf litter across five sites along an elevation gradient and (ii) a restoration experiment carried out at three sites. Cell counts were highly repeatable between the two technical replicates. For the restoration experiment samples (*n *= 90), the coefficient of variation (CV) of cell counts between the two technical replicates was only 6% but was 53% among the three biological replicates. Similarly, the CVs of cell counts among the technical and biological replicates in the elevation gradient samples (*n *= 30) were 4% and 19%, respectively. These results suggest that our analytical method adds little variation to the estimates of bacterial abundance relative to spatial variability among samples collected from different plots within a site.

**FIG 4:**
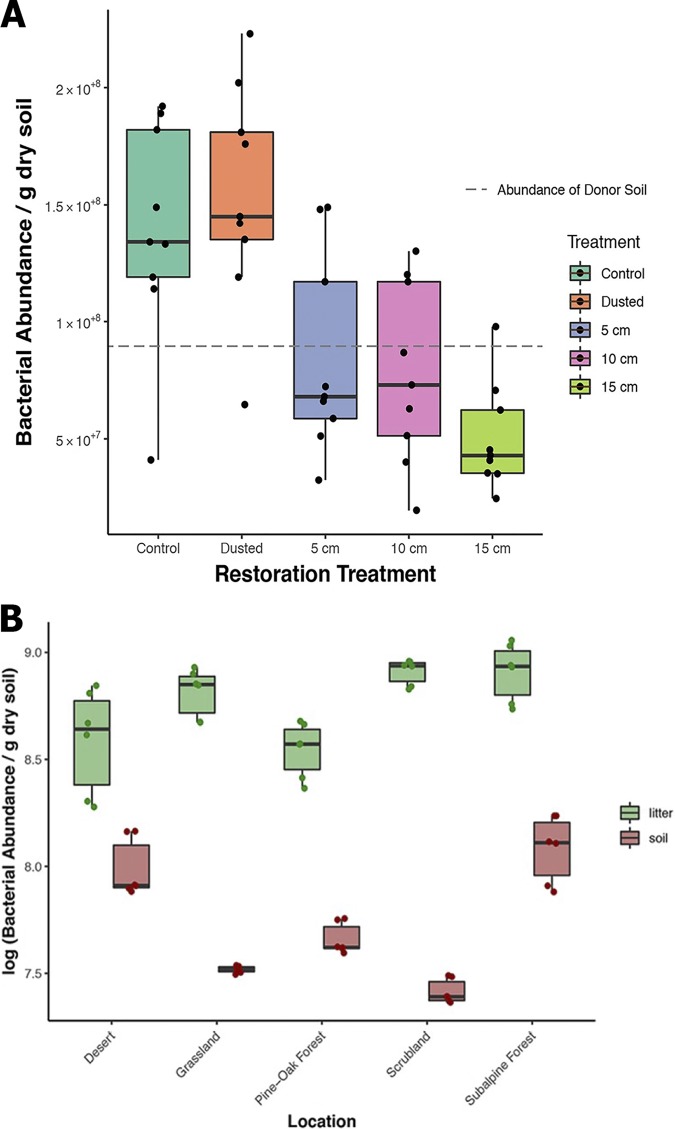
Bacterial abundances measured by flow cytometry (procedure C) in two field studies. (A) Bacterial abundances among the restoration treatments average across three experimental sites. The dashed line indicates the average bacterial abundance of the donor soil. (B) Bacterial abundances in surface leaf litter and bulk soil across five sites along an elevation gradient.

In the restoration experiment, bacterial abundance differed significantly between the soil application treatments (Fig. 4A) (two-way ANOVA, *F*_treatment_ = 9.90, *df* = 4, *P < *0.0001) but not among the three locations (*F*_site_ = 0.16, *df* = 2, *P *= 0.853). Bacterial abundances in the control plots and in those only lightly dusted with salvaged soil were similar, with averages of 1.39 × 10^8^ and 1.54 × 10^8^ cells/g dry soil in the control and dusted plots, respectively. In contrast, bacterial abundance decreased with the application of a 5-, 10-, or 15-cm-thick salvage soil layer, similarly to the average bacterial abundance of the donor soil (Fig. 4A) (8.95 × 10^7^cells/g dry soil).

Along the elevation gradient, bacterial abundance varied by substrate (bulk soil versus surface leaf litter) (two-way ANOVA, *F*_substrate_ = 52.085, *df* = 1, *P < *0.0001), ranging from 3.6 × 10^8^ g^−1^ dry litter in the pine-oak forest to 8.41 × 10^8^ g^−1^ dry litter in the subalpine forest (Fig. 4B), but not by site (*F*_site_ = 1.187, *df* = 4, *P *= 0.347). Cell abundances in bulk soil ranged over an order of magnitude, with the lowest counts in the scrubland site (2.6 × 10^7^ g^−1^ dry soil) and highest counts in the subalpine forest (1.3 × 10^8^ g^−1^ dry soil) (Fig. 4B). Abundances in bulk soil and leaf litter at each site were not correlated across habitats (*r* = −0.34; *P *= 0.21). Finally, percent carbon content and abundance were not correlated in either the soil or litter samples (*r*_soil_ = 0.34, *P *= 0.21; *r*_litter_ = −0.05, *P *= 0.85; *n *= 15).

## DISCUSSION

A variety of methods are available to quantify soil bacterial abundance, and previous studies have found that these methods are often repeatable and that their results correlated (4, 5). Still, it remains unclear what the ideal reference method should be, as all have known biases. Therefore, the goal of this study was to optimize the method further while quantifying its limitations. Using a density gradient extraction method, we improved on previously developed FCM protocols by maximizing the efficient cell recovery from soil while reducing interference from background debris. We achieved this by reducing the amount of sample used, adding a surfactant, and applying an intermediate degree of agitation through shaking. These procedures resulted in a high (89%) recovery rate of a positive addition (E. coli cells). Further, estimated abundances were highly repeatable across technical replicates (for the same sample), allowing us to detect significant differences in bacterial abundance across treatments in two field studies. While additional tests are needed, we conclude that current FCM methods to estimate bacterial abundance in field soils are reliable and sensitive.

Future work on the method can be divided into two categories. The first category is a further improvement of systematic biases or those that apply equally across samples and that therefore should not influence a study’s trends in abundance. For instance, we acknowledge that the recovery rate of a single pure culture is unlikely to accurately reflect the recovery of the diverse community of *in situ* bacteria. In particular, it may be easier to reextract the added cells than native cells, because the added cells do not have time to attach to soil particles. Thus, our estimates of percent recovery rates of native bacterial abundance are likely overestimates. A variety of cell extraction solutions have been used for soil FCM methods, including the dispersant sodium pyrophosphate (4), which is often mixed with a surfactant such as Tween 20 (20) or Tween 80 (21–23). Although we did not directly compare different combinations, we also found that a mixture of tetrasodium pyrophosphate (TSP) and Tween 80 resulted in high yields, supporting this idea. Our tests also supported the use of both chemical dispersants and mechanical treatment (sonication or shaking) (4, 22, 23), and sonication might be used to further reduce sample handling time (data not shown). In contrast, homogenization appeared to be too destructive as a mechanical treatment, as indicated by very low recovery of E. coli cells.

More problematic than a systematic bias in cell extraction is whether there are differential biases among soil types, the second category of future work on the application of FCM to soils. In the elevation study, cell recovery did not vary significantly by site but was positively correlated with percent carbon of the samples. This trend is opposite that previously reported, which suggested that high carbon content might reduce estimates of bacterial abundance, because higher total organic matter levels might make it difficult to distinguish cells from interfering particles (4). Other studies have also found that soil properties such as soil cation exchange capacity and soil organic matter content may bias FCM counts (24). Thus, more research is needed to test the extent of differential biases encountered in applying FCM protocols to soil samples that vary greatly in their physical characteristics.

Despite these biases, the soil and litter bacterial abundances that we reported from these southern California habitats fall within the range of those generally observed in terrestrial soils (4, 5, 7). We also detected higher bacterial abundance in litter than in surface bulk soil. We speculate that this pattern could be due to the larger amount and quality of organic matter in the litter, which provides more available substrates for the microbial activities (5, 15, 25). In addition, the smaller amount of clay particles might allow greater efficiency of cell extraction, which we did not test here. Notably, bacterial abundances in the litter and corresponding bulk soil were not correlated among the elevation gradient sites.

Our protocol also distinguished cell abundances among treatments in the restoration experiment and revealed biologically interpretable differences. For instance, the bacterial abundances in the control and dusted treatments were similar, as we expected. In contrast, the abundance in the treatments with thicker salvage soil applications resembled the donor soil, which was significantly lower. The use of salvaged topsoil has been observed to be more effective than other restoration methods at increasing native plant cover (26), and our results suggest that such treatments can alter bacterial abundances.

In sum, FCM seems to offer a direct, quick, and replicable approach to assay bacterial abundance in soil, but methodological concerns have inhibited its wider adoption. Here, we demonstrated how these concerns can be addressed quantitatively. While further tests of potential biases are needed across a broader range of environments, the method is sensitive enough to distinguish soil abundances among different treatments and environments.

## MATERIALS AND METHODS

### Study sites and field studies.

All samples (surface leaf litter and bulk soil) were collected from sites in southern California located on granitic parent material. The region experiences a Mediterranean climate, with hot, dry summers and cooler, wetter winters (15). Samples were transported in a cooler to the laboratory. For flow cytometry, 0.1 g of each sample was immediately fixed with 5 ml sterile saline solution (0.9% NaCl) containing 1% Pi-buffered glutaraldehyde. Fixed samples were stored at least overnight (and up to 30 days) at 4°C before processing.

### Restoration study.

For initial protocol testing, we collected soils from an experiment testing whether application of salvage soil to degraded sites improved plant community restoration. The soil donor site was located in Orchard Hills, Irvine (33°44′32.8″N, 117°44′14.7″W). In December 2015, soils were scraped 10 cm deep from 6 areas with native plants and cacti, combined, and transferred to three nearby sites (Hicks Haul [33°44′08.5″N, 117°42′25.1″W], West Loma [33°45′18.5″N, 117°44′28.0″W], and Portola Stage [33°41′54.6″N, 117°41′44.9″W]). At each recipient site, the same experimental design was implemented to test the effects of the depth of the donor soil on plant restoration outcomes (Table 2). Five treatments were applied in three replicate blocks. Within each block, five 16-m^2^ plots received one of each treatment: no added soil (control), a dusting of donor soil, or a 5-, 10-, or 15-cm-thick layer of donor soil. In January 2016, bulk soil samples were taken from each plot (a composite of three soil cores per plot, where each core was 2.5 cm in diameter and 10 cm deep) at the three recipient sites (*n *= 51). We also collected six samples from the donor site. The samples were returned to the laboratory, homogenized, and fixed for further analysis. Selected samples (*n *= 9) were used for initial protocol testing such that only 42 samples were analyzed with the optimized method. The chemical characteristics of soils from these sites were statistically similar (ANOVA, *P* > 0.05) (Table 2).

**TABLE 2:** Chemical characteristic of the soils at the four locations in the restoration study^*a*^

Site	pH	% C	% N
Orchard Hills	6.3 (0.08)	1.8 (0.8)	0.14 (0.06)
Hicks Haul	6.3 (0.04)	2.5 (0.9)	0.30 (0.16)
West Loma	6.0 (0.08)	3.0 (0.9)	0.33 (0.13)
Portola Stage	6.2 (0.07)	2.2 (0.14)	0.22 (0.00)

a Values in parentheses represent 1 standard deviation (*n *= 3).

### Elevation gradient study.

To test the optimized method across a range of soils, we collected leaf litter and surface bulk soil from five sites across an elevation gradient in Southern California (Table 3). The soils differ in temperature, precipitation, and plant community composition across the gradient (15). On 24 October 2016, bulk soil samples (composed of three cores, each 10 cm deep and 2.5 cm in diameter) and leaf litter samples (composed of a combination of three random handfuls) were collected from three 1-m^2^ plots at the sites for a total of 15 leaf litter and 15 bulk soil samples. The leaf litter samples were ground under sterile conditions using a bladed coffee grinder (model BCG111OB; KitchenAid, Benton Harbor, MI, USA).

**TABLE 3:** Descriptions of the ecosystems along the elevation gradient

Ecosystem	Latitude (N)	Longitude (W)	Elevation (m)	Total annual precipitation (mm)	Mean soil temp (°C)
Desert	33.648	−116.38	275	231.5	26.3
Scrubland	33.610	−116.45	1,280	428.4	17.4
Grassland	33.737	−117.70	470	569.4	18.8
Pine-Oak forest	33.683	−116.77	1,710	1,415.8	11.4
Subalpine forest	33.823	−116.75	2,250	1,376.5	11.0

A subsample of each bulk soil sample was air-dried. We used this sample to measure the pH of a 1:2 soil/water solution (Beckman Coulter, IN, USA) and total C and N with a Carlo Erba elemental analyzer (NA 1500 NC; Carlo Erba, Milan, Italy).

### Optimization of the cell extraction methods.

**FIG 5:**
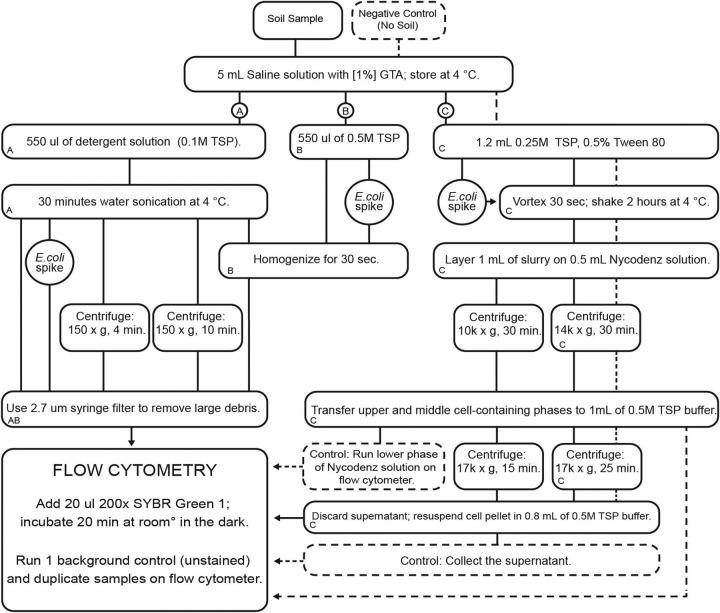
Work flow diagram for samples measured by flow cytometry in this study. The letters indicate the pathways of the three main protocols that were adjusted and compared by spiking samples with E. coli. The protocols used for controls are indicated by boxes composed of dashed lines. Additional comparisons of centrifugation speeds/timings within the main protocols are also shown, but they are not labeled with letters as they were not compared with E. coli spikes. GTA, glycerol triacetate.

We tested how signal detection and cell recovery were altered by three main procedures that used (i) an indirect ultrasonication bath and filtration, (ii) a tissue homogenizer and filtration, and (iii) shaking and Nycodenz density gradient separation pathways. Within these three methods, we also tested the use of different amounts of soil (0.1, 0.5, 1, and 2 g) (data not shown) and the effects of detergent concentration and the speed/duration of centrifugation (Fig. 5).

To test the effectiveness of the cell extraction protocol, a known number of Escherichia coli cells were added to a subset of the bulk soil samples. (This species was chosen because it is fast growing and easy to culture.) An E. coli culture was grown in LB media overnight at 37˚C, and its density was assessed on the flow cytometer. We diluted the culture with saline solution to equal 1.5 × 10^8^ cells/ml. We then added 20 μl of the solution (a total of 3 × 10^6^ cells) to each fixed soil sample (Fig. 5). The recovery efficiency was assessed as follws: (the number of cells in the E. coli spiked sample – the number of cells in the nonspiked sample)/the number of added E. coli cells.

The optimal extraction method (see Results), procedure C (Fig. 5), was applied to all samples (including bulk soil and leaf litter) from the two field studies. We combined 0.1 g of each sample with 1.2 ml of a detergent solution (250 mM tetrasodium pyrophosphate [TSP; pH 8.0] containing Tween 80 [0.5% final concentration]) and then added the E. coli solution. The solution was subjected to vortex mixing for 30 s and then shaken for 2 h at 4°C (Boekel Rocker II rocker, used at the highest setting with tilt angle of 20˚). After letting the soil slurry stand for at least 1 min, we slowly layered 1 ml of the soil slurry (in two technical replicates) onto 0.5 ml of Nycodenz solution (80% [wt/vol] prepared in 50 mM sterile TSP buffer), being careful not to mix the slurry and Nycodenz solution. Bacterial cells and soil particles were separated by high-speed centrifugation (14,000 × *g*) for 30 min. The upper and middle cell-containing phases (including the thin layer on top of the Nycodenz cushion liquid phase) were carefully recovered. The lower phase of the gradient was collected as a control to confirm that no cells were recovered (*n *= 2). The cell-containing phases were then mixed with 1 ml of the 50 mM TSP buffer and centrifuged at 17,000 × *g* for 25 min. The centrifugation produced a pellet containing the cell fraction. The supernatant was collected as a control to confirm that no cells remained (*n *= 4). Finally, the cell pellet was resuspended in 0.8 ml of the TSP buffer.

### Flow cytometry.

The technical replicates resulted in two final cell pellet slurries (400 μl), which were then stained with 2 μl of 200× SYBR green I (final SYBR green I concentration = 1×) and incubated for 20 min at room temperature in the dark. Flow cytometry was performed on either a BD 6C flow cytometer (BD Biosciences, San Jose, CA, USA) or a NovoCyte flow cytometer (ACEA Biosciences, San Diego, CA, USA). We used green fluorescence channel (FL1) and forward scatter (FSC-H) detectors to reduce the natural autofluorescence found in environmental samples.

Initial gates were based on a previous FCM study of bacteria on leaf litter (27). These gates were adjusted for the present study by comparing unstained and stained soil samples, including some samples spiked with E. coli, to identify the region of stained cells versus those representing background autofluorescence. A polygonal gate was delineated around the population of bacteria on a bivariate dot plot of log FL1 versus log forward scatter (Fig. 1).

The same gating strategy was applied to the two flow cytometers, and the same gates were used for all samples on the same machine to allow direct comparison between measured samples. When running the samples, we aimed for 500 to 2,000 events per s to avoid event overlap. We counted the number of events within the gated boundaries over 1 min for each sample. Counts were then averaged between the two technical replicates.

### Statistical analysis.

To test for differences in bacterial cell abundances in the field studies, ANOVA was performed using the aov function in R software environment 3.3.1 (R Development Core Team 2017). In the elevation gradient study, the effects of site and substrate and their interactions were tested. In the restoration study, the effects of site and salvage treatment and their interaction were tested. We used Pearson’s correlations on cube root-transformed data to test for a correlation between soil factors and cell recovery along the elevation gradient (*rcorr* function; Hmisc package R). All data are available at https://github.com/khalilib/mSphere.
